# Nanoscale Structural Modulation and Low-temperature Magnetic Response in Mixed-layer Aurivillius-type Oxides

**DOI:** 10.1038/s41598-018-19448-1

**Published:** 2018-01-17

**Authors:** Shujie Sun, Zezhi Chen, Guopeng Wang, Xiaoju Geng, Zhenyu Xiao, Zhuzhu Sun, Zhihu Sun, Ranran Peng, Yalin Lu

**Affiliations:** 1 Collaborative Innovation Center of Henan Province for Energy-Saving Building Materials, Xinyang Normal University, Xinyang, 464000 China; 2 CAS Key Laboratory of Materials for Energy Conversion, Department of Materials Science and Engineering, University of Science and Technology of China, Hefei, 230026 China; 3 Synergetic Innovation Center of Quantum Information & Quantum Physics, University of Science and Technology of China, Hefei, 230026 China; 4 National Synchrotron Radiation Laboratory, University of Science and Technology of China, Hefei, 230026 China

## Abstract

Nanoscale structural modulation with different layer numbers in layer-structured complex oxides of the binary Bi_4_Ti_3_O_12_-BiFeO_3_ system can give rise to intriguing phenomena and extraordinary properties, originating from the correlated interfaces of two different phases with different strain states. In this work, we studied the nanoscale structural modulation induced by Co-substitution in the Aurivillius-type oxide of Bi_11_Fe_3_Ti_6_O_33_ with a unique and naturally occurred mixed-layer structure. Nanoscale structural evolution via doping occurred from the phase-modulated structure composed of 4- and 5-layer phases to a homogeneous 4-layer structure was clearly observed utilizing x-ray diffraction and electron micro-techniques. Significantly, magnetic response for the samples under various temperatures was recorded and larger magnetic coercive fields (*e.g. H*_*c*_ ∼ 10 kOe at 50 K) were found in the phase-modulated samples. Analyses of the x-ray absorption spectra and magnetic response confirmed that the low-temperature magnetic behaviour should be intrinsic to the phase-modulated structure inside the structural transformation region, mainly arising from structural distortions at the correlated interfaces.

## Introduction

Oxide interfaces can easily influence electrical, magnetic and structural functionalities of the perovskite oxides, arising from chemical, strain, polarization discontinuity, and so on. Extensive previous studies have been focused on novel physical properties at interfaces in heterojunctions and superlattices, for instances, on exchange bias (EB), uniaxial magnetic anisotropy, quasi-two-dimensional electron gas (q2-DEG), etc^1–4^. Aurivillius-type layered complex oxides in Bi_4_Ti_3_O_12_-BiFeO_3_ system, potentially as the candidate to be single-phase multiferroics possessing electric dipole and magnetic ordering simultaneously at the room temperature (RT), recently have been attracting remarkable interests^5–9^. Such complex oxides with a general formula of Bi_*n*+1_Fe_*n*-3_Ti_3_O_3*n*+3_ are alternately stacked with two fluorite-like (Bi_2_O_2_)^2+^ layers and *n*-layer perovskite-like (Bi_*n-*1_Fe_*n*-3_Ti_3_O_3*n*+1_)^2−^ slabs^5^. This special layered architecture is similar to a ‘natural’ but ‘single phase’ superlattice and has many layered one-dimensional (1D) interfaces. Both experimental and theoretical studies have previously demonstrated that oxygen octahedral rotations, tilts and distortions at the interfaces account for newly occurred interface polar fields or interface magnetic modulations^10,11^. Therefore, studying interfaces of the layered perovskite oxides is an important and significant subject to either find a new material or understand the fundamental physics. If the ability to engineer the interfaces is available inside the layered structure, new fascinating phenomena could be discovered or a large enhancement of the resulting multiferroic performance could be realized.

The interface region of the Aurivillius-type layered oxides usually refers to the region of the nanostructure among the *n*-layer perovskite slabs, including one (Bi_2_O_2_)^2−^ layer and the two perovskite layers adjacent to the two sandwiching (Bi_2_O_2_)^2−^ layers. Aforementioned *n* indicates the number of perovskite layers per slab and usually takes an integer values, and the periodically repeating of the interface region constitutes the layered structure in normal layered complex oxides. In fact, in such periodically stacked layered structures, those with different integer *n* have been previously studied showing different physical properties, such as ferromagnetism, transition temperature and magnetoelectric effect^6,8,12^. Unfortunately, direct and deep modulating the interfaces in such periodically stacked structure is difficult, and therefore it is hard to introduce new physical properties via the direct modulation due to that the periodically stacked perovskite slabs are almost homogeneous with very similar interfacial strain states. Direct and minor interface modulation in the periodically stacked structures has been usually via the chemical modification, including doping alkaline-earth or rare earth metal ions into A-sites or substituting magnetic transition metal ions into B-sites inside the perovskite structure^13–15^. This direct modulation indeed could realize certain levels of multiferroic property enhancements, however, the symmetry of the periodic structural unit was not apparently broken in such modulations. In order to study interfacial structural distortions, the symmetry of the interfaces in the layered structure should be better largely broken by using different layer numbers of the perovskite slabs, which have been obviously done by using heterojunctions or superlattices in almost all previous studies. However, the use of either heterojunction or superlattice usually involves the complicated growth methods, and is a hetero-phase structure too. A new way of realizing a single phase with the largely broken structure, yet with a large modulation capability to the structure, would be much better to either as a new material finding or for further underline physics understanding.

Intriguingly, our previous work had reported a Bi_11_Fe_3_Ti_6_O_33_ (*n* = 4.5) material, a new naturally occurring mixed-layer single-phase compound, uniquely with different perovskite layers sandwiched by the (Bi_2_O_2_)^2+^ layers^16^. This material shows certain interesting enhancements over both ferroelectric and antiferromagnetic properties, however, a direct modulation to the structure and a large ferromagnetic enhancement were not realized yet. Based on the material’s phase-modulated structure, if a further modulation to this structure could be realized, the modulated structure could largely influence the structural deformation, electron density and magnetic structure, then further giving rise to some novel physical properties potentially. In the light of the above discussion, in this work, the naturally occurring mixed-layer structure of the Aurivillius-type oxide Bi_11_Fe_3_Ti_6_O_33_ was chosen and then a very minor Co-doping was adopted in this system to modulate their interfaces. This strategy can reveal potential via simultaneous chemically-driven symmetry controlling and structurally-driven strain controlling to give rise to novel structures and properties, which is very different from the doping in the homogeneous materials in previous works^13,15^. A structural transformation, gradually changing from the originally phase-modulated structure composed of both 4- and 5-layer phases to a new homogeneous 4-layer structure, was observed clearly for the first time. This nanoscale structural evolution inside the phase-modulated structure was visualized using the electron diffraction techniques. Anomalous low-temperature magnetic response was clearly observed and the correlation between the phase-modulated interfaces and the observed magnetic properties was discussed in details.

## Results and Discussion

### Nanoscale structural evolution

**Figure 1:**
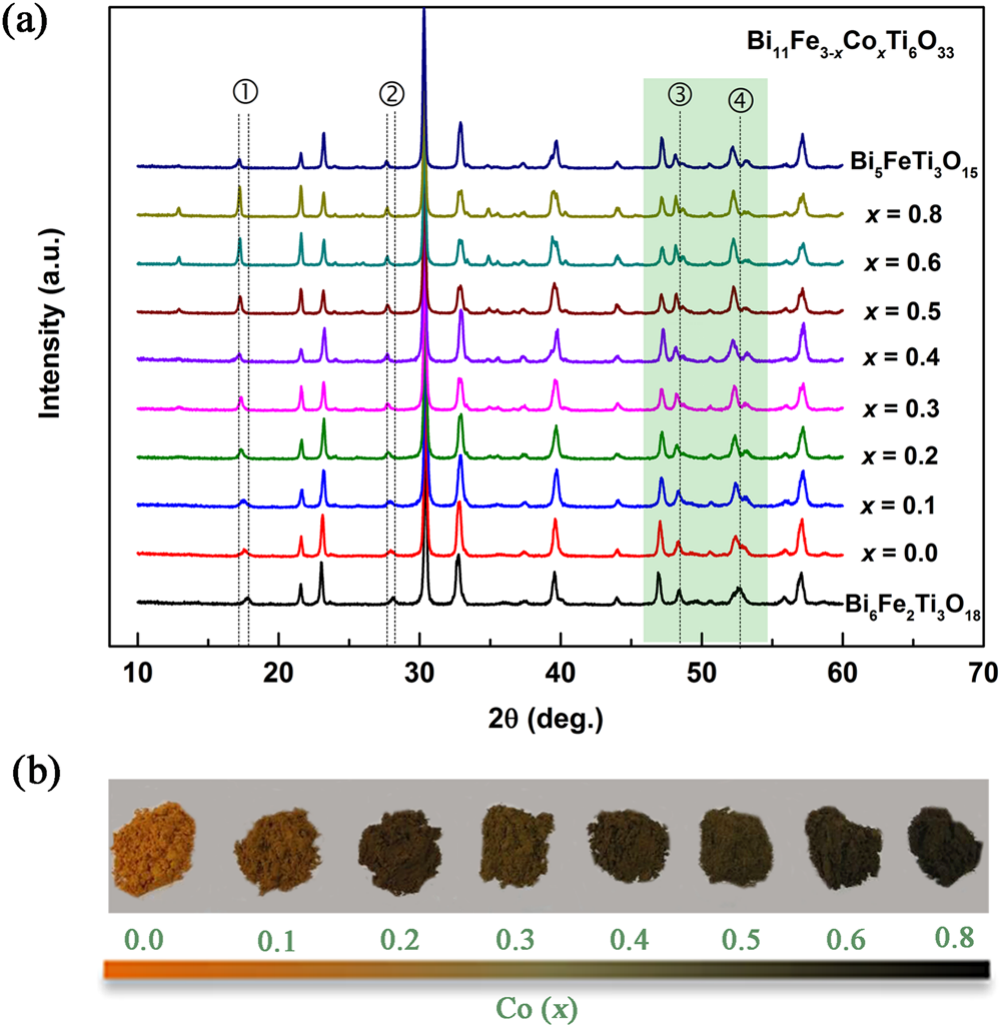
Structure evolution in BFCT-*x* and colour change of the powders. (**a**) Room temperature x-ray diffraction patterns of BFCT-*x*, Bi_5_FeTi_3_O_15_ (*n* = 4) and Bi_6_Fe_2_Ti_3_O_18_ (*n* = 5) powders; (**b**) The optical photos of the BFCT-*x* powders.

XRD patterns of BFCT-*x*, Bi_5_FeTi_3_O_15_ and Bi_6_Fe_2_Ti_3_O_18_ powders at the RT are shown in Fig. 1(a). All diffraction peaks were identified as belonging to the Aurivillius-type phase, implying that the amount of impurity phases, if exist, is below the XRD’s instrumental resolution. In contrast to the XRD of related reference samples, some large peak position shifts were observed when increasing the Co content. Especially, for BFCT-*x* (*x* = 0.0 ∼ 0.3), the peaks occurring at 17.2°∼17.8° and 27.7°∼28.2° (corresponding to ➀ and ➁ in Fig. 1(a), respectively) shift toward the lower angle, and the peaks at ∼48.4° and ∼ 52.6° (corresponding to ➂ and ➃ in Fig. 1(a), respectively) both gradually split into two peaks when increasing the amount of Co. However, when *x* ≥ 0.4, the observed XRD peaks for BFCT-*x* are nearly invariable and match to those from 4-layer Bi_5_FeTi_3_O_15_ (JCPDS, no. 82–0063). The XRD Rietveld refinements (see Supplementary Fig. S1) suggest that the observed XRD peaks match well to those of Bi_5_FeTi_3_O_15_ (*F2mm*) when *x* = 0.4 and of Bi_5_FeTi_3_O_15_ (*A2*_1_*am*) when *x* = 0.8. These results imply that Co substitution may lead to a reduction in layer number (*x* = 0.0∼0.3) and a transformation of the space group (*x* = 0.4∼0.8), of which the former can be obviously reflected in the peaks of (00 *l*) occurred at 12°–22° (Supplementary Fig. S2), and the latter indicated by the evolution of (200) and (020) peaks (Supplementary Fig. S3). The dependence of the lattice constant on the Co content *x* is shown in Supplementary Fig. S4. Variation of the lattice constant as *x* is distinct in phases with mixed-layer and four-layer numbers. It can be clearly seen that all the parameters in the mixed-layer structure reduce with an increase of the Co content and a split of *a* and *b* in the four-layer structure supports *F2mm* to *A2*_1_*am* phase transition. In short, above XRD results indicate an existence of structural transformation with a wide range in BFCT-*x*, gradually changing from the originally phase-modulated structure composed of 4- and 5-layer phases to a homogeneous 4-layer structure. Our previous work^16^ had proved that the BFCT-0.0 has the phase-modulated mixed-layer structure, characterized by disordered intergrowths of the 4- and 5-layer perovskite slabs. Qualitative XRD analyses suggest that Co substitution may lead to a reduction in the amount of the 5-layer perovskite slabs, thus cause nanoscale structural evolution of the mixed-layer structure in the transformation region. In addition, the minor amount of Co content in the samples significantly influences the colour of the powders, as shown in Fig. 1(b). The colours graded gradually from orange to black when increasing the doping *mol* amount of Co, probably implying a gradually widening visible-light absorbance^17^.

**Figure 2:**
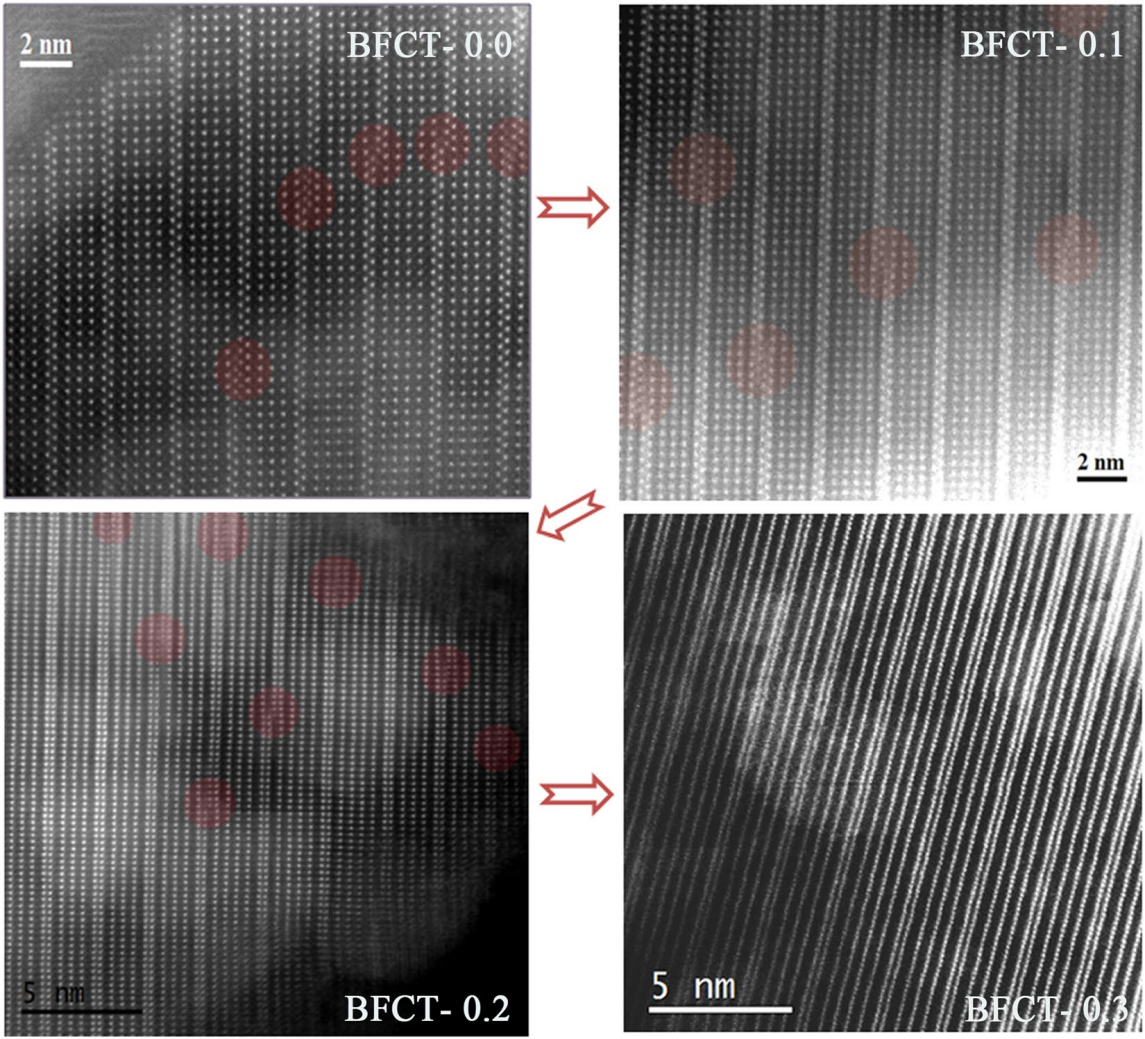
STEM-HAADF images of BFCT-0.0, 0.1, 0.2 and 0.3. The orderly arranged bright spots and the red circles represent bismuth atoms and positions of the defect, respectively.

To make the atomic structure visualized, the state-of-the-art STEM-HAADF images of BFCT-*x* (*x* = 0.0, 0.1, 0.2 and 0.3) were obtained, as shown in Fig. 2. The orderly arranged bright spots all belong to the Bi atoms in all images. Three or four layers of Bi atoms sandwiched by two closely stacked Bi layers [namely the (Bi_2_O_2_)^2−^ fluorite-type layer] can be easily understandable as corresponding to 4- or 5-layer perovskite slabs, and the Ti/Fe/Co atoms are neatly arranged between the three or four layers of Bi atoms. Observably, the images show a typical Aurivillius-type mixed-layer structure characterized by disordered intergrowths of the 4- and 5-layer perovskite slabs, which are well consistent with the aforementioned XRD results. For BFCT-0.0, the amount of the 5-layer perovskite slabs in the mixed-layer structure is more than that of the 4-layer slabs, while this is completely opposite in BFCT-0.3. For BFCT-0.1 and BFCT-0.2, they both presents that the amount of the 4-layer slabs is slightly more than that of the 5-layer slabs, and the latter is more significant. This means that lots of the interfaces of the 4- and 5-layer perovskite slabs are existed in BFCT-0.1 and BFCT-0.2. In addition, the mutual change of the 4- and 5-layer perovskite slabs can generate from a special shift (similar to a kind of point-defects) at some fluorite-type (Bi_2_O_2_)^2−^ layers, seeing the red circles in Fig. 2. These special shifts or the interfaces between the 4- and 5-layer slabs may easily result in complicated strains and distortions. In brief, the STEM-HAADF images suggested that the change of the ratio of the 4- and 5-layer slabs or a reduction in the 5-layer slab numbers is an essence of the evolution of one-dimensional (1D) phase-modulated structure during the structural transformation region.

### X-ray absorption

**Figure 3:**
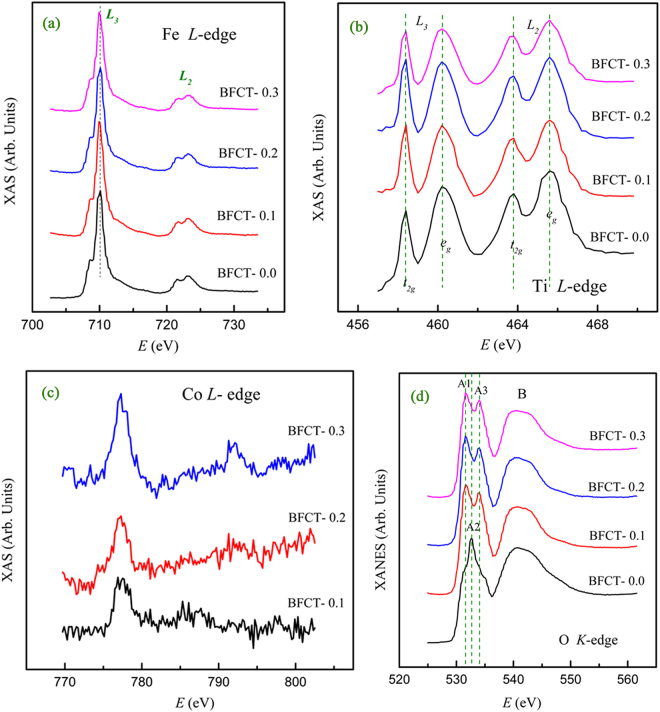
XAS spectra of BFCT-*x*. (**a**) Fe L-edge; (**b**) Ti L-edge; (**c**) Co L-edge; (**d**) O K-edge.

X-ray absorption (XAS) spectroscopy is sensitive to determine the charge state, structural environment and electronic structure of materials in an atom-specific manner. We present XAS spectra for the Fe L-edge, Ti L-edge, Co L-edge and O K-edge in BFCT-*x* (*x* = 0.0, 0.1, 0.2 and 0.3), as shown in Fig. 3. The Fe L-edge absorption spectra of BFCT-*x* are plotted in Fig. 3(a). Normally, because of Fe: *2p* spin-orbit coupling, the overall spectral shapes are similar for all iron species with an intense peak at around 708∼710 eV (L_3_) and a less intense peak at 721∼723 eV (L_2_). While the L_3_ and L_2_ peak positions do not differ significantly for Fe^2+^ and Fe^3+^, the fine structure is sensitive to the local environment of the absorber^18^. Our Fe L-edge XAS spectra exhibit fine structure at both the L_3_ and L_2_ edges, in contrast to the L-edge spectra for metallic Fe, where any fine structure is absent^19^. The first peak (L_3_) estimated at ∼710.1 eV, which is differ to the position of the α-Fe_2_O_3_ (Fe^3+^, ∼710.8 eV) and the FeTiO_3_ (Fe^2+^, ∼709.3 eV)^19^, indicating that the majority of Fe is not present as a secondary phase of α-Fe_2_O_3_ or FeTiO_3_ in the BFCT-*x*. The fine structure of the Fe L-edge spectra suggests a ‘+3’ valence of iron in the BFCT-*x* materials. The low-energy shoulders of the L_3_ and L_2_ peaks of Fe^3+^ are usually taken as indications of a high-spin state of iron in the crystal field of the oxygen ligand^19^. The very similar spectral shapes of the Fe L-edge spectra in samples indicate that the minor Co-doping causes no obvious changes on structural environment or electronic structure of Fe ions. The Ti L-edge absorption spectra of BFCT-*x* are shown in Fig. 3(b). Because titanium is dominated by the dipole *2p-3d* transition, spin-orbit splitting of the *2p* orbitals into *2p*_3/2_ (L_3_) and *2p*_1/2_ (L_2_), and crystal field splitting of the *3d* orbitals into *e*_*g*_ and *t*_*2g*_, result in a manifold of four absorption peaks for the Ti L-edge^18^. If the tetragonal distortion from perfect octahedral symmetry exists, the *e*_*g*_ states will be further split, *e.g*. anatase and rutile, where the intensity ratio of the split *e*_*g*_ peaks is opposite for the two crystal structures^20^. Compared the observed Ti L-edge XAS spectra with that of TiO_2_^18^, Ti should be a ‘+4’ valence and octahedrally coordinated by oxygen in BFCT-*x*.

Figure 3(c) shows the Co L-edge spectra of BFCT-*x*. The Co signals are rather weak despite the use of the total electron yield mode to collect the spectra, due to the very small Co concentrations. In contrast to the Co L_2,3_-edge spectra from the ordered and the disordered region in the La_2_CoMnO_6_ film^21^, the spectral shapes from BFCT-*x* are close to those from the disordered region, suggesting the existence of intermediate valence states for cobalt in BFCT-*x*. This result implies the mixed-layer structure may be similar to one of the disordered regions. Figure 3(d) shows the normalized O K-edge XANES spectra of BFCT-*x*. Strikingly, the spectral features of BFCT-0.0 are quite different from those of BFCT-*x* (*x* ≠ 0). Two strong pre-edge peaks A1 and A3 are present for BFCT-*x* (*x* ≠ 0), similar to those of Bi_6_FeCoTi_3_O_18_ and LaBi_5_FeCoTi_3_O_18_ thin films^22^, but appear only as two shoulders for BFCT-0.0. Moreover, the strong peak A2 appearing in the spectrum of BFCT-0.0 could be reduced in height as the result of Co substitution for Fe, even at the low Co concentrations. Distinctly different XANES spectral behaviours of the Co-doped and un-doped samples demonstrate that the local electronic structures of BFCT-0.0 are modified by Co-substitution. To the pre-peaks in the O K-edge, titanium oxides usually consist of two strong pre-edge peaks arising from transitions from O 1 *s* to hybridized Ti *3d* - O *2p* orbitals and crystal field splitting of the Ti *3d* orbitals into *e*_*g*_ and *t*_*2g*_ components^18^, yet iron (cobalt) oxides have only one pre-edge peak originating from transitions from O 1 *s* to hybridized Fe (Co) *3d* - O *2p* orbitals^19,23^. It is reasonable to understand that the change of the concentration ratio between Ti and Fe (Co) in the unit of BFCT-*x* gives rise to this different O K-edge XANES spectral behaviours, corresponding to the aforementioned structural transformation from the dominant 5-layer slabs (Ti/Fe ∼ 3/2) to the homogeneous 4-layer slabs (Ti/Fe ∼ 3/1). The main absorption edge (peak B) is assigned to the transitions from O 1 *s* to hybridized Ti 4*sp* - O *2p*, Fe *4p* - O *2p*, Co *4p* - O *2p* and Bi 6 *s* - O *2p* states^24,25^.

### Raman analysis

**Figure 4:**
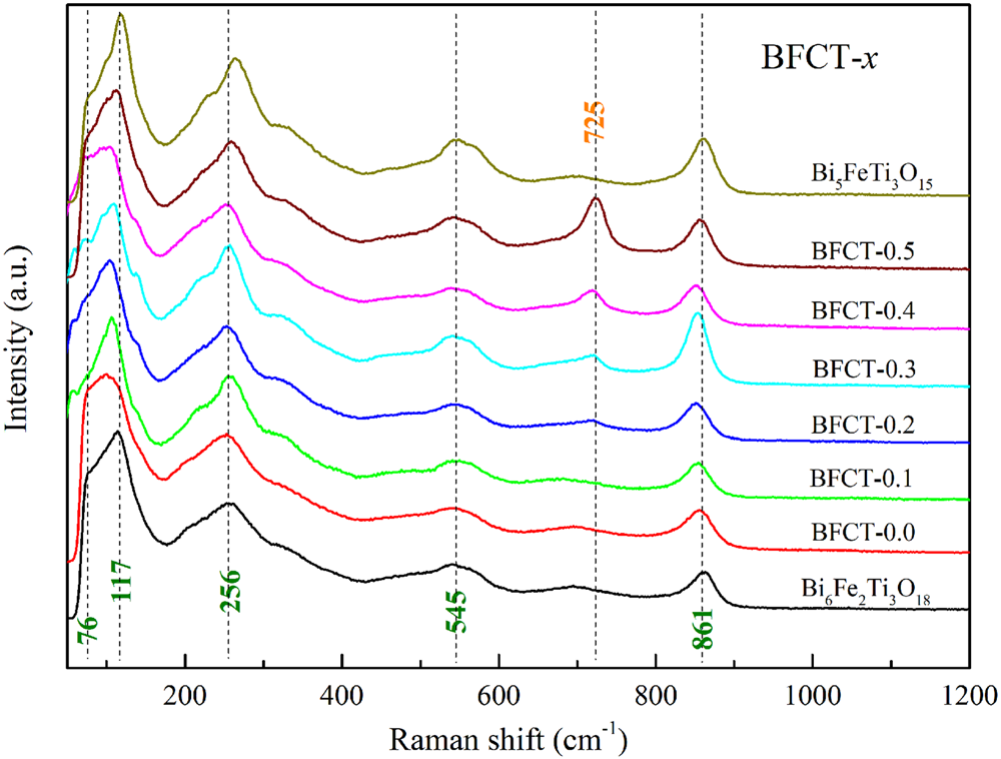
Raman spectra of the BFCT-*x* at room temperature.

Raman spectra of the BFCT samples were investigated in the frequency range of 50–1500 cm^−1^, as shown in Fig. 4. Normally, for the Raman spectrum of bismuth layer-structured crystals, the phonon modes below 200 cm^−1^ is ascribed to the vibration Bi^3+^ ions at (Bi_2_O_2_)^2−^ layers or perovskite slabs, and the phonon modes above 200 cm^−1^ result from bend and stretch of octahedral BO_6_^26^. In Fig. 4, the modes below 200 cm^−1^ of the BFCT (*x* = 0.1 ∼ 0.3), *e.g*. 76 cm^−1^ and 117 cm^−1^, show obvious differences when comparing with those of the BFCT-0.0, Bi_5_FeTi_3_O_15_ and Bi_6_Fe_2_Ti_3_O_18_. Our previous work had reported that this change below 200 cm^−1^ may originate from different strain states in the mixed-layer structure^16^, which affects the vibration of the Bi^3+^ ions at the A-sites in different perovskite layers^27^. This suggests the existence of the structural distortions at the interface of the two phases in the mixed-layer samples. To the best of our knowledge, the structural distortions mainly belong to three types: oxygen octahedral rotations, Jahn-Teller distortions and bending distortions, which are strongly related to polarization and magnetization^27^. It should also be noted that a new mode at ~ 725 cm^−1^ appears and increases substantially with the increase of the Co content. This mode may be caused by the vibration of the CoO_6_ octahedra, which can be observed in the Raman spectra of LaCoO_3_ and La_0.8_Sr_0.2_CoO_3_^28^. This result clearly indicates that the Co ions have entered the perovskite slabs. In addition, the major modes at about 256 cm^−1^, 545 cm^−1^ and 861 cm^−1^, which can be ascribed to O-Ti-O bending, Ti-O torsional bending and the symmetric stretching of the TiO_6_ octahedra, respectively^26^, do not shift obviously with the increase of the Co content. This result may suggest that cobalt atoms replace the iron atoms, rather than titanium atoms in the structures.

### Magnetic response

Magnetic properties of BFCT-*x* were carefully examined at the RT and at the high temperatures, as shown in Supplementary Fig. S5. Excitingly, the magnetization (*M*) increases when increasing Co content, and all samples exhibit a weak ferromagnetic (FM) nature. Remnant magnetization (2*M*_*r*_) and saturation magnetization (*M*_*s*_) increases as the doping level of cobalt increases, except for a small enhancement occurring at the transformation region (seeing Supplementary Fig. S6). Interestingly, magnetic coercive field (2*H*_*c*_) of BFCT-0.1 and BFCT-0.2 is greater than that of any other samples, suggesting a large interaction between anti-ferromagnetic (AFM) and FM domains. In order to exhibit superior RT magnetic property of the materials with the mixed-layer structure, we also had compared the RT *M-H* Loop of BFCT-0.2 with that of two references samples, homogeneous phase Bi_5_Fe_0.8_Co_0.2_Ti_3_O_15_ (4-layer) and Bi_6_Fe_1.8_Co_0.2_Ti_3_O_18_ (5-layer), as shown in Supplementary Fig. S5. Significantly, under the same doping level of cobalt, the BFCT-0.2 has better magnetic response than the reference samples, whether 2*M*_*r*_, *M*_*s*_ or 2*H*_*c*_. Zero-field-cooled (ZFC) and field-cooled (FC) curves for the BFCT-*x* (Supplementary Fig. S6) exhibit a feature of FM state and undergo an FM-paramagnetic (PM) transition. The magnetic Curie temperature (*T*_*C*_) increases firstly and then decreases when increasing the Co content and the largest *T*_*C*_ is ∼786 K for BFCT-0.2.

**Figure 5:**
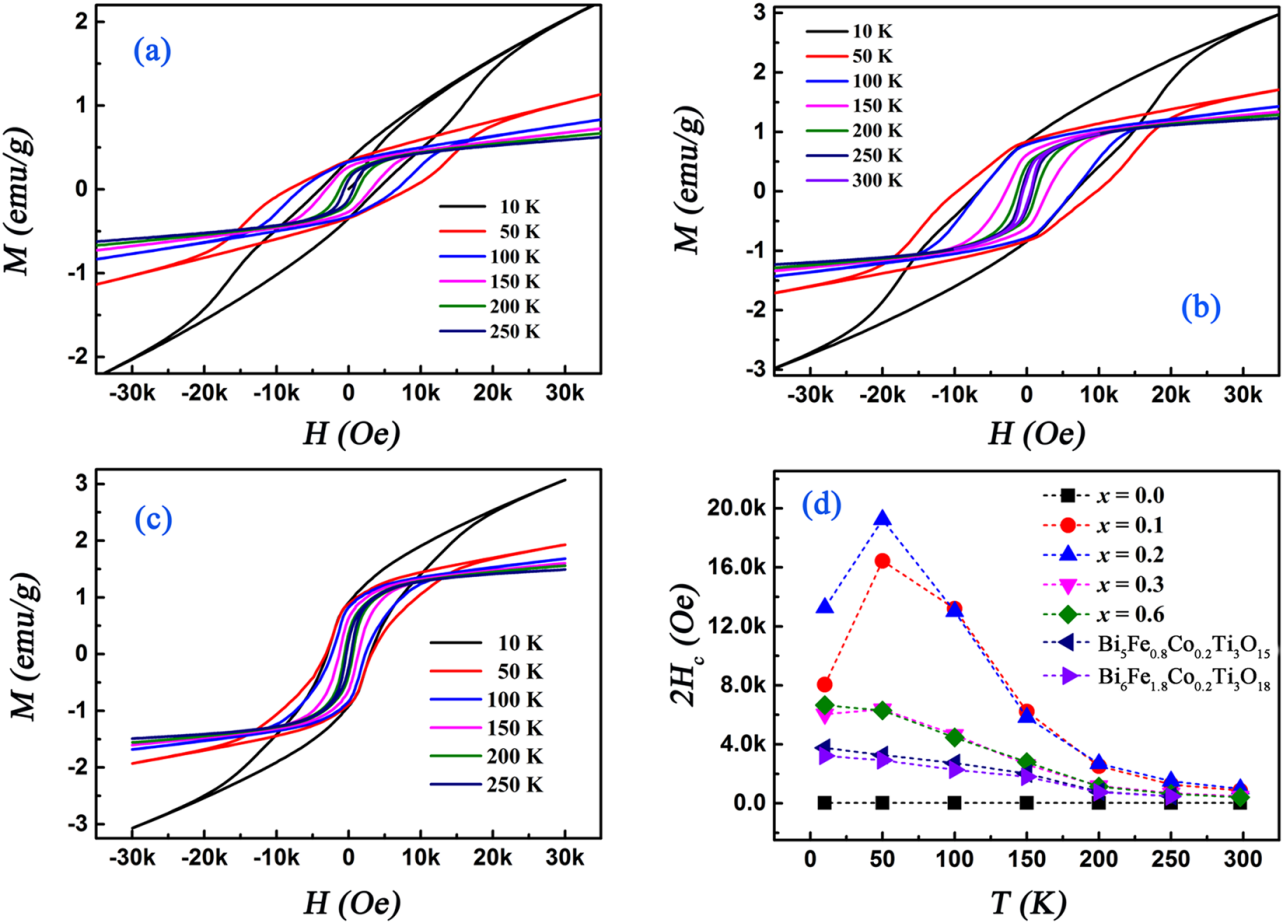
Low-temperatures magnetic property of BFCT-*x*. Hysteresis loops of (**a**) BFCT-0.1, (**b**) BFCT-0.2 and (**c**) BFCT-0.3 at different low-temperatures with measuring field between ± 40 kOe. (**d**) Temperature dependence of 2*H*_*c*_ for BFCT-*x* (*x* = 0.0, 0.1, 0.2, 0.3 and 0.6), Bi_5_Fe_0.8_Co_0.2_Ti_3_O_15_ (*n* = 4) and Bi_6_Fe_1.8_Co_0.2_Ti_3_O_18_ (*n* = 5). The latter two were as the reference samples.

In order to take into account the nature of magnetic structure and make clear the correlations between magnetic and structural properties, we performed the magnetic hysteresis measurements at different low temperatures, as shown in Fig. 5. In addition, in order to distinguish the influence of secondary FM inclusions, we also observed the *M-H* curves of three FM materials (spinel-phase pure CoFe_2_O_4_ ceramic, predesigned ZrO_2_-13weight% CoFe_2_O_4_ composite ceramic and BiFe_0.7_Co_0.3_O_3_ ceramic) at different low temperatures, as shown in Supplementary Figs S7–S9. Obviously, the *M-H* plots of three FM materials show a common feature that 2*H*_*c*_ increases monotonically while *M*_*S*_ decreases monotonically when lowering temperature. Excitingly, the *M-H* plots of BFCT-0.1 [Fig. 5(a)], BFCT-0.2 [Fig. 5(b)] and BFCT-0.3 [Fig. 5(c)] present a completely different trend. *M*_*S*_ increases monotonically while 2*H*_*c*_ increases firstly and then decreases when decreasing temperature. This phenomenon strongly supports that the observed *M-H* curves are the intrinsic magnetic responses and not the contributions of the secondary FM inclusions. The large 2*H*_*c*_ for BFCT-*x* (*e.g*. 2*H*_*c*_ ∼ 20 kOe for BFCT-0.2 at 50 K) further verifies intrinsic magnetic behaviour. The reasons are as follows: 1) derivative thermo-magneto-gravimetric (DTMG) technique^29^ found no CoFe_2_O_4_-type impurity in the mixed-layer samples, as shown in Supplementary Fig. S10; 2) the minor CoFe_2_O_4_ impurity could be unable to exhibit so much 2*H*_*c*_; 3) if any, the mainly magnetic contribution should be from the minor CoFe_2_O_4_ impurity, so the 2*H*_*c*_ at 10 K would be higher than the value at 50 K, but the fact is just not the case. In Fig. 5(a–c), we found the *M-H* plots clearly exhibit a typical FM behaviour above 100 K, while a main AFM features below 50 K, suggesting an existence of the competition between AFM and FM interaction. Significantly, at 10 K, a larger hysteresis loop and unsaturated magnetization even at high fields can be clearly observed in all samples, revealing of a superposition of both AFM and FM components and indicating strongly AFM behaviour at lower temperatures. The temperature dependence of 2*H*_*c*_ for BFCT-*x* is shown in Fig. 5(d). Normally, 2*H*_*c*_ for a ferromagnet decreases monotonically when increasing temperature, arising from thermal activities. The similar trend in BFCT-0.6 indicates the samples with excessive Co content show a main FM behaviour. However, for BFCT-0.1, BFCT-0.2 and BFCT-0.3, 2*H*_*c*_ increases firstly and then decreases as rising the temperature, indicating an existence of anomalous magnetic behaviour in the mixed-layer samples. The change of 2*H*_*c*_ in BFCT-0.1 and BFCT-0.2 is more significant that in BFCT-0.3, suggesting that anomalous magnetic response may be correlated with the coexistence of 4- and 5-layer perovskite slabs and specifically the interfaces of them. The values of 2*H*_*c*_ in BFCT-0.1 and BFCT-0.2 at 50 or 100 K are all above 10 kOe and even some values can reach∼20 kOe, which are far higher than those in BFCT-0.3 and 0.6 (about 4 ∼ 6 kOe). To the best of our knowledge, the order of magnitude of 2*H*_*c*_ in 10∼150 K is even comparable with that of Fe-Co alloys^30^, cobalt ferrite nanoparticles^31^, and hole-doped La_1−*x*_Sr_*x*_CoO_3_ (∼10 kOe at 5 K)^32^.

The ZFC-FC curves for BFCT-*x* in the region 10–300 K under 100 Oe are shown in Supplementary Figs S11 and S12. For BFCT-0.0, the ZFC-FC curves exhibit a characterization of PM-like state, implying dominant AFM behaviour. For BFCT-0.1 and BFCT-0.2, the ZFC-FC curves are different from those for BFCT-0.0, suggesting the coexistence of AFM and weak FM behaviours at very lower temperatures. When *x* ≥ 0.3, the large space between ZFC and FC curves below 150 K indicates an existence of the strongly FM state at very lower temperatures. Dong *et al*.^33^ reported the long-range magnetic orderings were no existence inside Aurivillius-type multiferroic oxides and pointed out that locally Fe-O-Fe clusters would result in AFM interaction. Zhao *et al*.^34^ suggested short-range magnetic orderings form in iron-rich areas in Aurivillius-type multiferroic thin films, which would cause the FM behaviour or spin/cluster glass behaviour. Accordingly, large FM moment in the minor Co-doped samples may originate from Fe^3+^-O-Co^3+^ or Fe^3+^-O-Co^2+^ exchange interaction in iron-rich areas^33–35^ and spin canting *via* anti-symmetric Dzyaloshinskii-Moriya interaction^7,36^. Based on the discussions of the XRD, XAS and Raman results, the anomalous magnetic response in BFCT-*x*, especially BFCT-0.1 and BFCT-0.2, might be explained as follows: 1) the mixed-layer structure and point defects at (Bi_2_O_2_)^2−^ layers could result in distortions nearby special interfaces of two different perovskite slabs, especially bending and Jahn-Teller distortions, which are easily generated at interfaces of the two different phases with different strain states and strongly correlated with the magnetic transition^3,11^; 2) the mixed-layer structure could give rise to many disordered regions and structural distortions, and parts of Fe (Co) ions may be likely to form clusters in the host lattice, which contribute to the short-range FM ordering. As a result, it is an existence of strongly magnetic interaction between intrinsic AFM ordering and short-range FM ordering. When *x* ≥ 0.3, the disordered structure turns into the ordered structure and the Co contents gradually increase, and thus Co ions may be deemed as a random distribution or uniform distribution in materials system. The magnetic interactions reflect in the long-range magnetic ordering, showing a main FM characteristic.

**Figure 6:**
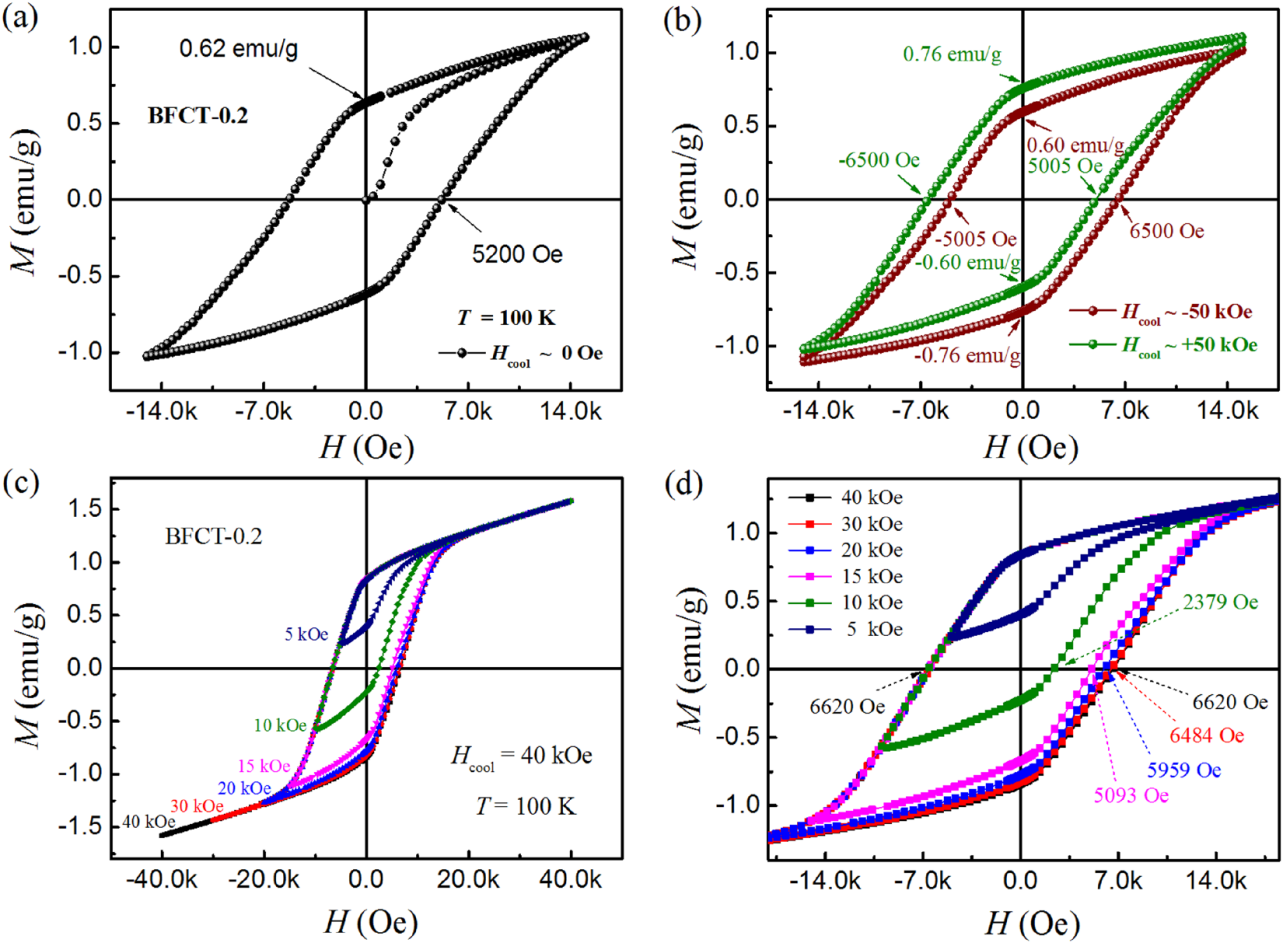
(**a**,**b**) Hysteresis loops of BFCT-0.2 at 100 K with both the ZFC and FC processes. For the ZFC/FC process, the sample was cooled in zero/±50 kOe magnetic field from 380 to 100 K, and then the hysteresis loops were measured between ±15 kOe. (**c**) The field-cooled (FC, in 40 kOe) hysteresis loops for BFCT-0.2 at 100 K with different measuring magnetic fields. (**d**) The magnified view of the *M-H* curves in “(**c**)”.

Exchange bias phenomenon is often observed in many different systems containing FM/AFM interfaces. We measured the hysteresis loops of BFCT-0.2 at 100 K with both the ZFC and FC processes. For the ZFC/FC process, the sample was cooled in zero/±50 kOe magnetic field from 380 to 100 K, and then the hysteresis loop was measured between ±15 kOe, as shown in Fig. 6(a) and (b). The *M*_*ZFC*_*-H* loop has a normal hysteresis loop centred at zero field, but the *M*_*FC*_*-H* loop (in +50 kOe) shifts both to the negative field and to the positive magnetization, and the shifts of the *M*_*FC*_*-H* loop (in −50 kOe) are completely opposite. The gravity centre offset is the same to the *M*_*FC*_*-H* loop for the FC process cooled in ± 50 kOe. However, the above observation does not support the existence of the exchange-bias effect, because the appearance of both horizontal and vertical shifts is the fact that the hysteresis loops of the sample measured at low temperature are unsaturated, i.e., minor hysteresis loops which are shifted along both field axes and magnetization axes^37^. To confirm this claim, we studied the influence of measuring field on the hysteresis loop in BFCT-0.2 at 100 K. The sample was cooled in a field of 40 kOe from 380 to 100 K, then the hysteresis loops were measured between 40∼ −5 kOe, 40∼ −10 kOe, 40∼ −15 kOe, 40∼ −20 kOe, 40∼ −30 kOe and 40∼ −40 kOe, respectively, as shown in Fig. 6(c) and (d). When the measuring field is low, especially *H* ≤ 10 kOe, the unsaturated *M-H* loops always are clearly shifted along both the negative field and the positive magnetization axis directions. But when the measuring field is high enough, *H* ≥ 40 kOe, the *M-H* loop become a saturated magnetically hysteresis loop and do not show any shift. The characteristics of these loops, which are similar to those of a ferromagnet, have nothing to do with the exchange-bias phenomenon and the shifts are naturally explained by the fact that the loops are unsaturated. Therefore, its well-known manifestation that the *M-H* loop shift away from the zero field axis should be estimated by measuring the coercive fields of the descending and ascending branches of the loop, respectively, of a saturated magnetically hysteresis loop^38^. The exchange-bias phenomenon is not observed in our samples with the strongly AFM/FM interaction, and the reason might be from weak or nonexistent AFM/spin glass (SG) interaction, compared to the reported Aurivillius-type oxides which have the exchange-bias effect due to the coupling between AFM and SG^8,39^.

## Conclusions

In summary, we firstly observed nanoscale structural evolution in BFCT-*x* by presenting XRD patterns and STEM-HAADF images. A reduction in 5-layer perovskite slab numbers is an essence of the structural evolution of the phase-modulated phases during the structural transformation region. Magnetic measurements under different temperatures show that anomalous magnetic response, especially larger 2*H*_*c*_, only exists in the phase-modulated sample. The change of the *M-H* plots of the BFCT-*x* when decreasing the ambient temperature is completely different from that of the FM materials, confirming intrinsic magnetic behaviour. The analyses of the magnetic properties, Raman and XAS results proved that the magnetic response in the BFCT-*x* arises from the intrinsic 1D phase-modulated structure and the use of minor Co-doping, which could easily generate structural distortions at interfaces of two different phases thus give rise to some weak FM regions forming strong FM/AFM interaction.

## Methods

Bi_11_Fe_3-*x*_Co_*x*_Ti_6_O_33_ (BFCT-*x*, *x* = 0.0~0.8) powders were synthesized using the modified Pechini’s method. Fabrication of the BFCT-*x* ceramics follows the procedures of pre-sintering for powders and then hot-press sintering of the pellets for ceramics. Detail synthesis conditions and procedures were similar as our previous report^16^. As reference samples, Bi_5_FeTi_3_O_15_ (4-layer, *n* = 4), Bi_5_Fe_0.8_Co_0.2_Ti_3_O_15_ (4-layer, *n* = 4), Bi_6_Fe_2_Ti_3_O_18_ (5-layer, *n* = 5) and Bi_6_Fe_1.8_Co_0.2_Ti_3_O_18_ (5-layer, *n* = 5) were also synthesized using the same method. Phase structures of the samples were investigated by x-ray diffraction (XRD) patterns recorded on an x-ray diffractmeter employing Cu-K*α* radiation (Rigaku TTR III). Layer-structures of the samples were visualized by the aberration corrected scanning transmission electron microscopy high angle annular dark field (STEM-HAADF) images carried out on a scanning transmission electron microscope (JEM-ARM200F). Magnetic properties were characterized by using a vibrating samples magnetometer (VSM) option of the Quantum Design Physical Property Measurement System (PPMS) at the different temperatures. Raman spectra were measured with a Laser Raman spectrometer using 514.5 nm line of an Ar +laser as excitation source (LabRamHR). Fe, Ti, Co L-edges and O K-edge x-ray absorption (XAS) spectra were measured at the BL12B-a beamline of National Synchrotron Radiation Laboratory (NSRL, Hefei, P. R. China) using the synchrotron radiation from the storage ring running at 800 MeV with an average current of 300 mA.

## Electronic supplementary material

Supplementary Info
