# Supplementary material for: Nanoscale Structural Modulation and Low-temperature Magnetic Response in Mixed-layer Aurivillius-type Oxides
Source: Sci Rep. 2018 Jan 17;8:871. doi: 10.1038/s41598-018-19448-1 (PMC5772624; doi:10.1038/s41598-018-19448-1)
Supplement: Supplementary file 1 — Supplementary Info [file 41598_2018_19448_MOESM1_ESM.pdf]

Electronic Supplementary Information

Nanoscale Structural Modulation and Low-temperature Magnetic Response in Mixed-layer Aurivillius-type Oxides

Shujie Sun,[ab*] Zezhi Chen,[b] Guopeng Wang,[b] Xiaoju Geng,[a] Zhenyu Xiao,[a] Zhuzhu Sun,[a]

Zhihu Sun,[d] Ranran Peng [bc*] and Yalin Lu [bcd*]

[a] *Henan Collaborative Innovation Center of Energy-Saving Building Materials, Xinyang Normal University, Xinyang 464000, China*

[b] *CAS Key Laboratory of Materials for Energy Conversion, Department of Materials Science and Engineering, University of Science and Technology of China, Hefei 230026, China*

[c] *Synergetic Innovation Center of Quantum Information & Quantum Physics, University of Science and Technology of China, Hefei 230026, China*

[d] *National Synchrotron Radiation Laboratory, University of Science and Technology of China, Hefei 230026, China*

* Corresponding Authors: ***sjsun@xynu.edu.cn; pengrr@ustc.edu.cn; yllu@ustc.edu.cn***

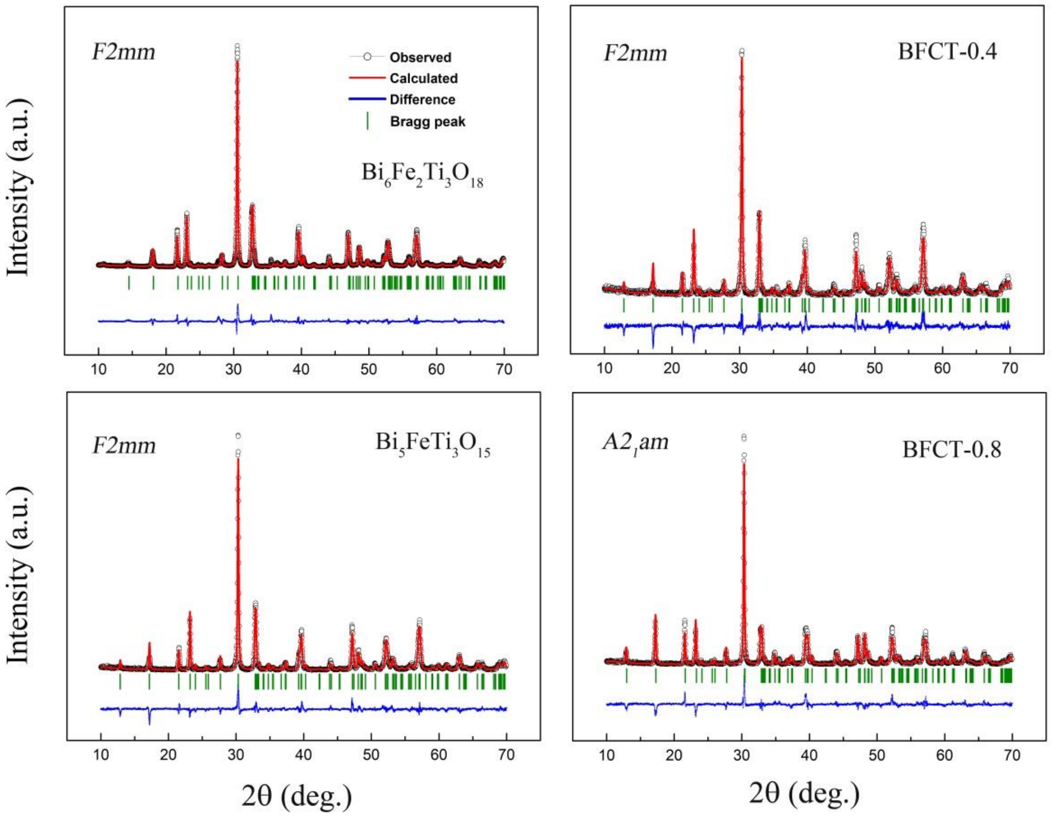

Fig. S1 Rietveld refinements for the powder XRD patterns of the sintered $Bi_6FeTi_3O_{18}$, BFCT-0.4, BFCT-0.8 and $Bi_5FeTi_3O_{15}$ ceramics. These XRD patterns were refined in the orthorhombic lattice using GSAS software. Circles indicate the experimental data and calculated data are the continuous red line overlapping them. The blue curve shows the difference between the experimental and calculated patterns. The vertical bars indicate the expected reflection positions. The lattice parameters and space groups turn out to be a = 5.461 Å, b = 5.472 Å, c = 49.388 Å for $Bi_6FeTi_3O_{18}$ (*F2mm*, wR_p = 4.2%), a = 5.438 Å, b = 5.434 Å, c = 41.275 Å for BFCT-0.4 (*F2mm*, wR_p = 5.4%), a = 5.440 Å, b = 5.442 Å, c = 41.211 Å for $Bi_5FeTi_3O_{15}$ (*F2mm*, wR_p = 6.8%) and a = 5.463 Å, b = 5.437 Å, c = 41.156 Å for BFCT-0.8 ($A2_1am$, wR_p = 7.1%).

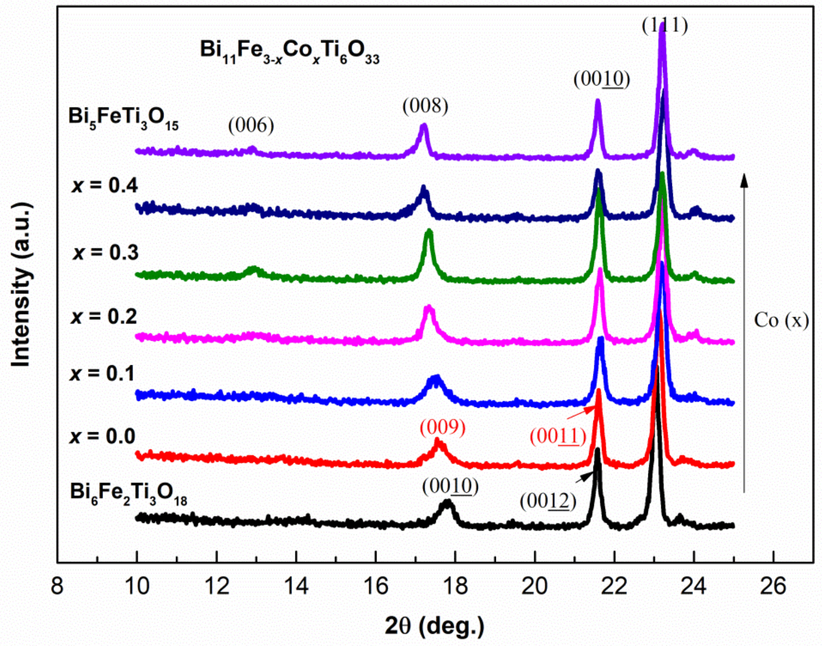

Fig. S2 Selected section of the XRD patterns of the BFCT-*x* in the 2θ range of 10°-25°. Based on the orthorhombic lattice, we can easily calculate to lattice parameter *c* by the reflections (*00l*). Obviously, lattice parameter *c* is gradually diminishing with increasing the amount of Co ($x = 0.0 \sim 0.4$).

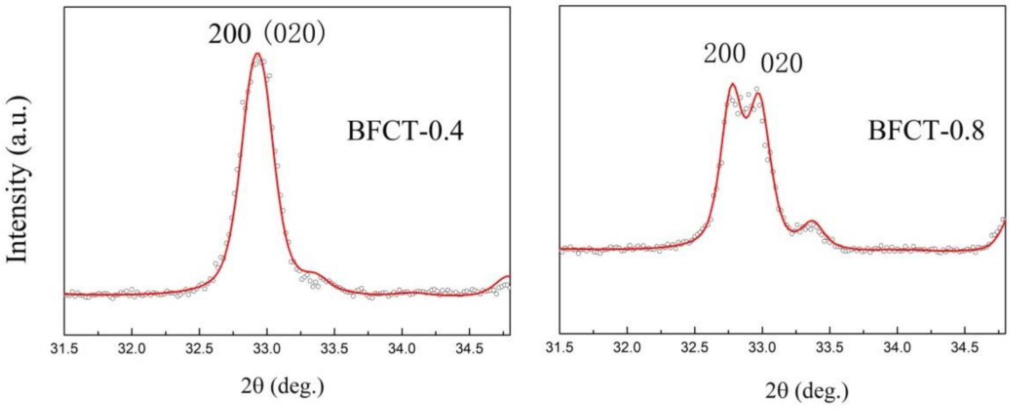

Fig. S3 The evolution of the XRD peaks (200) and (020). The two peaks begin to overlap each other when $x = 0.4$ and then are separated with increasing the amount of Co (*for example, BFCT-0.8*). This result supports the transformation of space groups ($F2mm \rightarrow A2_1am$).

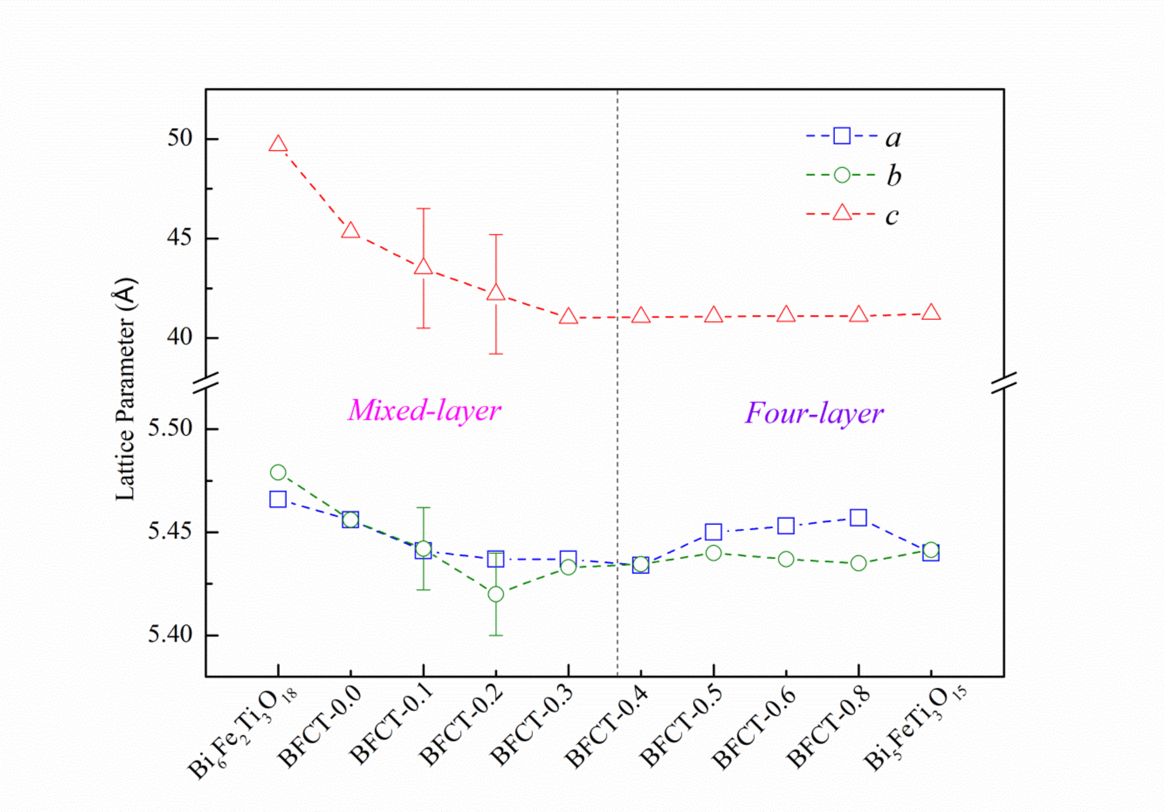

Fig. S4 Lattice constants of BFCT-*x* with various Co content refined by the Rietveld method. These XRD patterns were refined in the orthorhombic lattice using GSAS software and JADE 5. For BFCT-0.1 and BFCT-0.2, there are no suitable crystal structures (CIF format files). The calculated results are from the average values by using $Bi_{11}Fe_3TI_6O_{33}$.cif and $Bi_5FeTi_3O_{15}$.cif. The width of the error bar is 3Å for *c* and 0.02 Å for *a* and *b*.

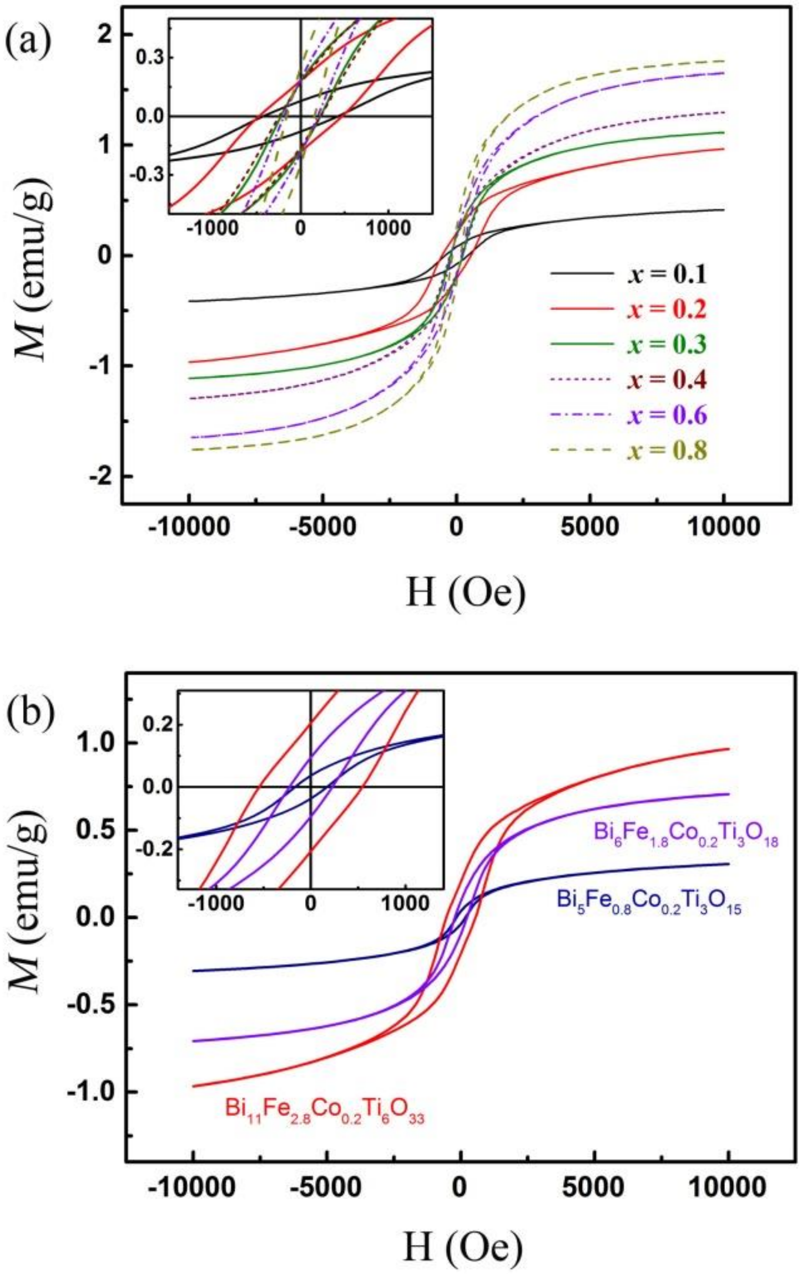

Fig. S5 Room-temperature magnetic property of BFCT-*x*. (a) Magnetic hysteresis loops of the samples at RT. The inset shows the magnified view of the M-H curves. (b) The M-H loops of $Bi_5Fe_{0.8}Co_{0.2}Ti_3O_{15}$, $Bi_{11}Fe_{2.8}Co_{0.2}Ti_6O_{33}$ (BFCT-0.2) and $Bi_6Fe_{1.8}Co_{0.2}Ti_3O_{18}$ at RT. The inset shows the magnified view of the M-H curves.

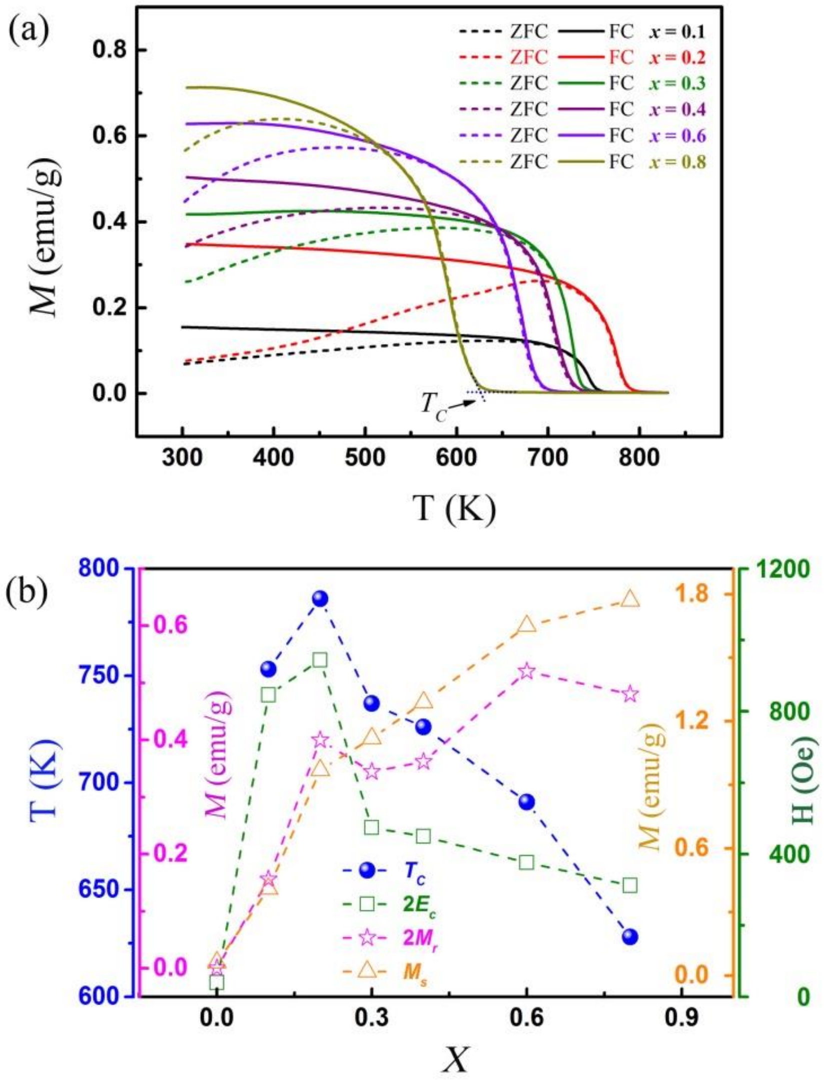

Fig. S6 High-temperature magnetic property of BFCT-*x*. (a) Temperature dependence of magnetization for the samples in the zero-field-cooled (ZFC) and field-cooled (FC) modes under a magnetic field 500 Oe. All the samples undergo a ferromagnetic (FC) - paramagnetic (PM) transition and the magnetic Curie temperature (T_C) was defined as the temperature corresponding to the inflection point of the *M*-*T* curves. (b) Remnant magnetization ($2M_r$), saturation magnetization (M_s), magnetic coercive field ($2H_c$) and T_C vs. the doping level of Co (*x*).

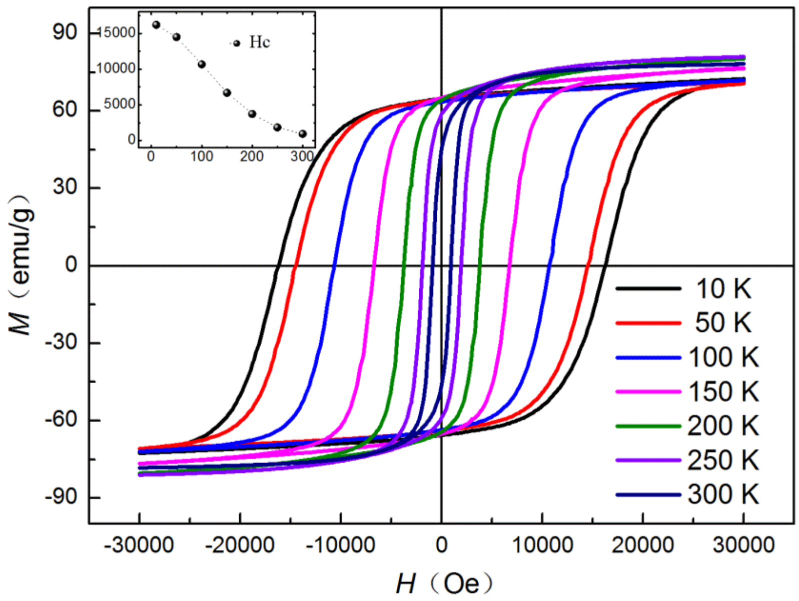

Fig. S7 Hysteresis loops of $CoFe_2O_4$ ceramic at different temperatures. The inset shows the temperature dependence of H_c for $CoFe_2O_4$. Spinel-phase $CoFe_2O_4$ is usually deemed to a FM secondary phase from Co-substituted Aurivillius-type multiferroic oxides.

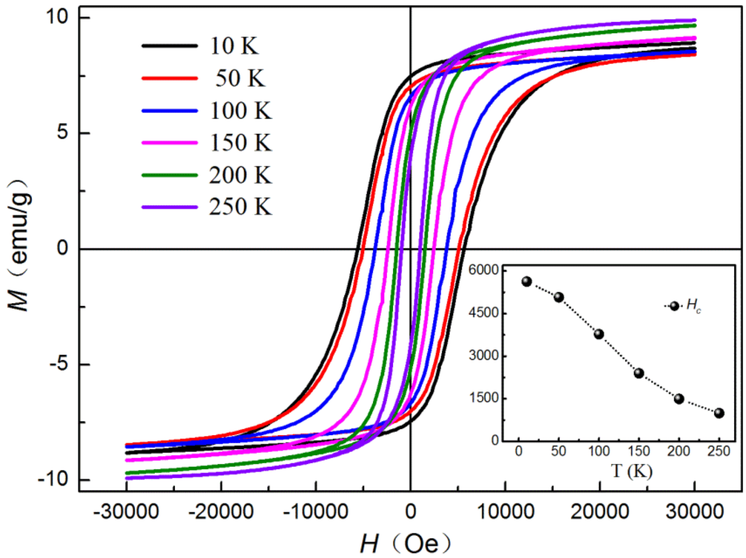

Fig. S8 Hysteresis loops of ZrO_2-13weight%$CoFe_2O_4$ ceramic at different temperatures. The inset shows the temperature dependence of H_c for ZrO_2-13weight%$CoFe_2O_4$. Since ZrO_2 is not a magnetic material, magnetic response of ZrO_2 -13 weight%$CoFe_2O_4$ should be derived from a small amount of the $CoFe_2O_4$ phase.

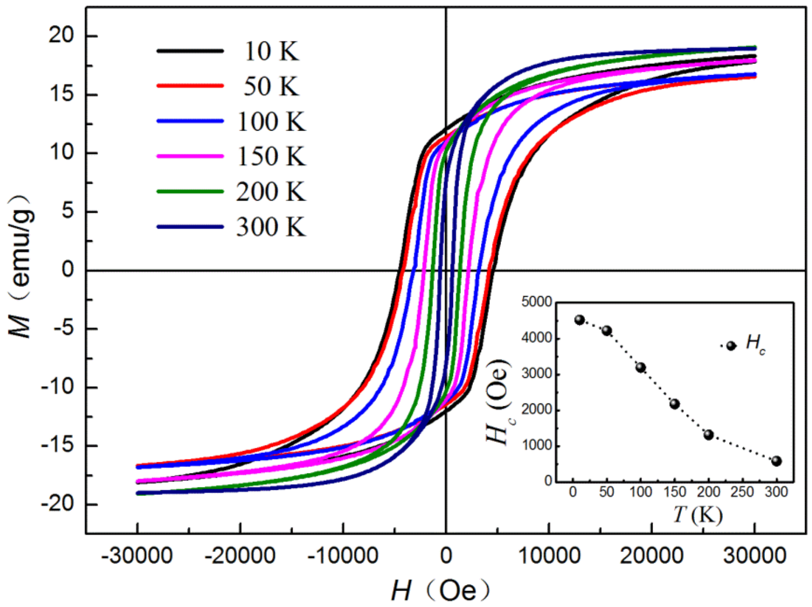

Fig. S9 Hysteresis loops of $BiFe_{0.7}Co_{0.3}O_3$ ceramic at different temperatures. The inset shows the temperature dependence of H_c for $BiFe_{0.7}Co_{0.3}O_3$. $BiFe_{0.7}Co_{0.3}O_3$ might be a FM secondary phase formed during the sample preparation and its magnetic property is better than that of the rest of $BiFe_{1-x}Co_xO_3$. Obviously, the M-H plots of the above three FM materials (Fig. S7-S9) show a common feature that $2H_c$ increases monotonically while M_S decreases monotonically when lowering temperature.

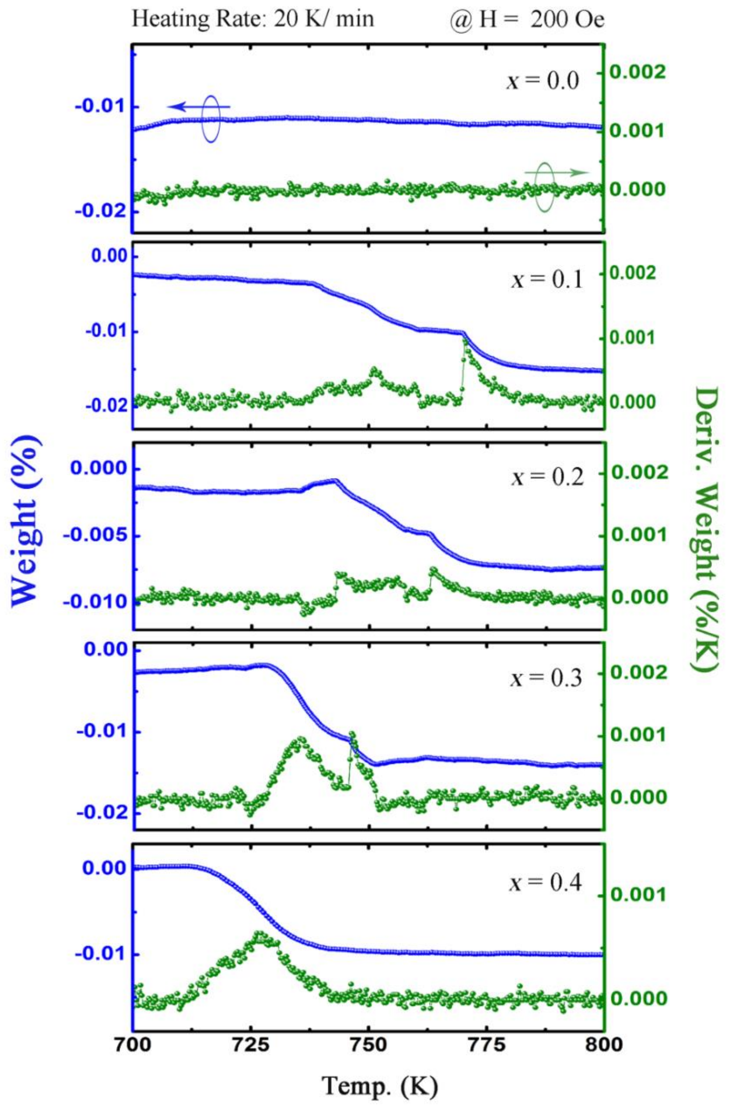

Fig. S10 Weight loss and derivative thermo-magneto-gravimetric (DTMG) curves of BFCT-*x* (x = 0.0, 0.1, 0.2, 0.3 and 0.4), performed in a nitrogen atmosphere with a heating rate of 20 K/min and an applied magnetic field of 0.02 T. Under a magnetic field, weight loss from the contribution of thermally active regions has been automatically deducted. We had reported that a ferromagnetic-to-paramagnetic transition peak for pure $CoFe_2O_4$ occurs at a temperature of ∼720 K in the derivative weight (dW/dT) curve in Ref. 7. According that the accuracy of this DTMG measurement experimentally reaches to ~0.5 weight.% [Ref. 29], the figure implies that the amount of $CoFe_2O_4$-type phase, if exist, is below ~0.5 weight.%. Two peaks for BFCT-0.1, 0.2 and 0.3 in the dW/dT curves come from the co-existent and nanoscale structurally modulated five- and four-layer phases.

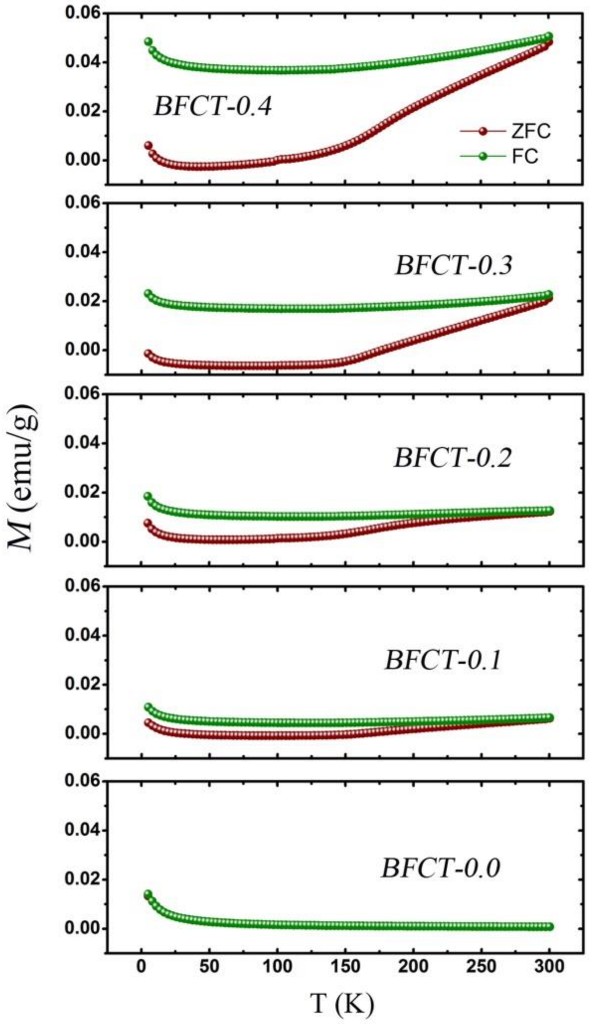

Fig. S11 The temperature dependence of magnetization with the ZFC and FC processes for BFCT-*x* (*x* = 0.0, 0.1, 0.2, 0.3 and 0.4) was measured in the region 10-300 K under 100 Oe. For BFCT-0.0, the ZFC-FC curves exhibit a characterization of PM-like state, implying dominant AFM behaviour. For BFCT-0.1 and BFCT-0.2, the ZFC-FC curves are different from those for BFCT-0.0, suggesting the coexistence of AFM and weak FM behaviours at very lower temperatures. When $x \geq 0.3$, the large space between ZFC and FC curves below 150 K indicates an existence of the strongly FM state at very lower temperatures.

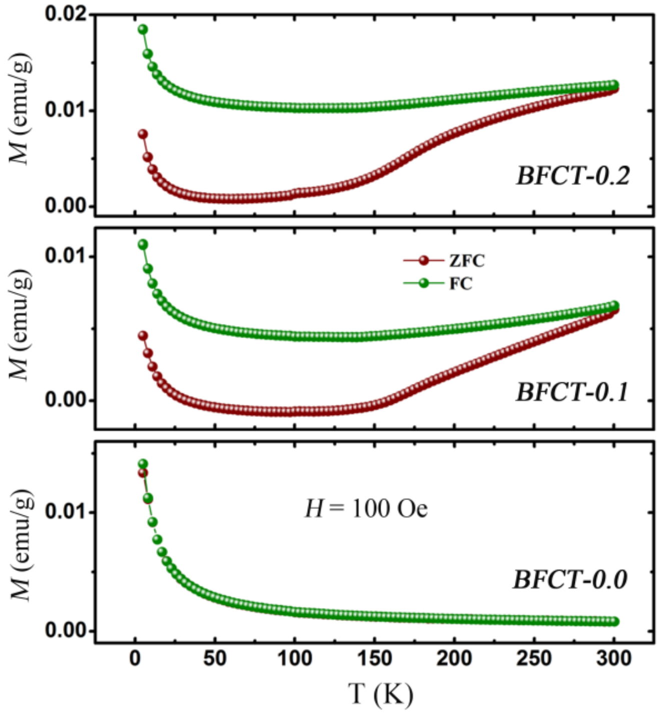

Fig. S12 ZFC-FC curves for BFCT-0.0, 0.1 and 0.2 in the region 10-300 K under 100 Oe. For BFCT-0.1 and 0.2, the deviation between ZFC and FC begins to occur at about 300 K, where is the measured temperature boundary, The ZFC magnetization undergoes a less rapid drop down to 150 K, and then remains nearly invariant value in the region 50~150 K, finally, it rises sharply below 50 K. The FC magnetization undergoes a comparatively less rapid change down to 50 K and then rises sharply below 50 K.
